# Motivation and Barriers for Seeking Osteoporosis Screening Services Among the Elderly Population in Al-Madina Munawara

**DOI:** 10.7759/cureus.63225

**Published:** 2024-06-26

**Authors:** Heba A Nawaz, Asmaa Alqusibri

**Affiliations:** 1 Madinah Health Cluster, Saudi Ministry of Health, Madinah, SAU

**Keywords:** barriers, al-madina munawara, bone mass, motivation, osteoporosis

## Abstract

Background

Osteoporosis is a chronic bone disease associated with a reduction in bone mass and an increased risk of fractures. The prevalence of osteoporosis is rising globally, including in Saudi Arabia, where there is a lack of information regarding the uptake of osteoporosis screening services. This study aims to examine self-efficacy and barriers toward osteoporosis screening in older women and men in Al-Madina Munawara, Saudi Arabia.

Methods

A cross-sectional study was conducted among adults aged 60 and above who attended primary healthcare centers. Convenience sampling was used to recruit participants, and a self-administered questionnaire was used to assess sociodemographic characteristics, osteoporosis status, general health-related characteristics, and screening self-efficacy. Analyses included multivariable regression analyses to evaluate the association between osteoporosis screening self-efficacy and potential explanatory variables. Data were collected in the last quarter of 2023.

Results

In a study involving 342 completed questionnaires, the mean age of participants was 66.2 years (SD = 4.3), with a range from 60 to 79 years, and the majority were male (230, 67.3%), having chronic diseases (226, 66.3%). Regarding osteoporosis risk factors and screening behaviors, the majority did not use prednisolone (252, 74.1%), did not have a family history of osteoporosis (216, 63.2%), had not experienced falls in the past five years (223, 65.2%), and had not undergone osteoporosis screening (299, 87.4%). The mean self-efficacy score for osteoporosis screening was 37.7 (SD = 4.7), ranging from 10 to 50, which indicated a moderate level of screening self-efficacy. In multivariate analysis, smokers were more likely to have higher scores in self-efficacy for osteoporosis screening compared to non-smokers (RR = 1.10; 95% CI = 1.01, 1.21). Participants who did osteoporosis screening (RR = 1.12; 95% CI = 1.01, 1.24) and those who were planning to do osteoporosis screening (RR = 1.10; 95% CI = 1.03, 1.19) were more likely to have higher score in self-efficacy for osteoporosis screening compared to their counterparts.

Conclusion

The participants had a fair level of screening self-efficacy. The smokers and those who had undergone or planned to undertake osteoporosis screening demonstrated higher self-efficacy scores than others. A lot of progress could be made in decreasing the burden of osteoporosis and enhancing the overall health and well-being of the older population by addressing these issues using specific interventions and policy measures.

## Introduction

Osteoporosis is a chronic bone disease characterized by a reduction in bone mass, leading to an increased risk of fractures [1,2]. It is a silent epidemic that progresses slowly and often manifests asymptomatically until bone fractures occur, particularly among women and the elderly [3]. By 2025, it is projected that there will be over 3 million fractures annually worldwide, resulting in a total cost of $25.3 billion [4]. Asian women, due to their unique body weight and anatomical characteristics, are more prone to decreased bone mineral density and an elevated risk of osteoporosis. It is predicted that by 2050, Asia will account for more than half of the global incidence of hip fractures [5]. The prevalence of osteoporosis in Saudi Arabia is reportedly increasing, with an estimated rate of 58.4% among women aged 50-80 years and 63.6% among healthy men [6]. Despite adequate sunlight, there is a widespread occurrence of vitamin D deficiency among both children and adults in Saudi Arabia [7]. This situation can be partially attributed to genetic variations and the cultural practice of clothing that covers the skin, limiting sunlight exposure [8]. Preventive measures, including lifestyle modifications, a healthy diet, and screening, can effectively reduce the disabilities and complications associated with osteoporosis [9]. Screening should involve assessing risk factors for falls, conducting a bone density test, and considering other possible secondary causes of osteoporosis [10].

In Saudi Arabia, the prevalence of osteoporosis is significant, with 30.7% of males and 34% of women aged 50-79 affected [11]. It is estimated that Saudi Arabia experiences approximately 8,768 femoral fractures each year, resulting in substantial costs and indicating the significance of this public health issue [12]. In Saudi Arabia, the Saudi Osteoporosis Society (SOS) has established guidelines for the management of osteoporosis, which encompass behavior change, diagnosis, screening, and treatment [13]. However, there is a lack of information regarding the uptake of osteoporosis screening services in the country. Awareness of the importance of preventive measures and early detection is a motivator for osteoporosis screening. Individuals with a higher level of knowledge and understanding about osteoporosis are supposed to be more likely to recognize the value of screening services and proactively seek them out. One study conducted in Saudi Arabia found that more than one-third of the participants had moderate or poor knowledge of osteoporosis [14]. Increased education and awareness campaigns can contribute to improving this motivator and consequently enhance screening uptake. This highlights the importance of exploring the barriers and motivations to screening services among Saudis, in order to develop strategies for improving the services and increasing the uptake. This, in turn, would effectively manage osteoporosis within the population. Studying osteoporosis screening uptake in Saudi Arabia lies in the significant burden of the disease, the unmet need for screening utilization, and the potential benefits of increased screening uptake. Investigating self-efficacy, barriers to screening, and designing effective interventions can improve the early identification of at-risk individuals, enhance patient outcomes, reduce healthcare costs, and contribute to the overall improvement of public health. This study aimed to examine self-efficacy about osteoporosis screening in older women and men in Al-Madina Munawara, Saudi Arabia, to determine barriers to screening.

## Materials and methods

This is an analytic cross-sectional study conducted in Al-Madina Munawara, which included adults aged 60 and above attending primary healthcare centers (PHCs) in Al-Madina. The recruitment was done according to SOS recommendations and willingness to participate in the study after obtaining informed consent.

The sample size was calculated using OpenEpi.com with an error of 5% and 95% CI, and the expected percentage of moderate to poor knowledge was 33.3% [15]. The estimated sample size was 311 patients. In addition, we added 10% to compensate for non-responders; the final sample size was 342 patients. A multistage random sampling technique was used as follows. Stage 1: we got a list of all PHCs in Al-Madina (urban region), which was a total of 53 PHCs. Stage 2: we selected eight PHCs by using a simple random sampling method. Stage 3: within the selected PHCs, patients were selected using a systematic random sampling method. Those who met the inclusion criteria and provided informed consent were included in the study. Participants meeting the inclusion criteria for this study were required to be visitors of PHCs, aged 60 years or older, and willing to provide informed consent. Exclusion criteria were applied to individuals who refused to complete the questionnaire, submitted incomplete responses, had a healthcare background, or were aware of osteoporosis cases. These criteria aimed to ensure that the study selected a sample of older adults from the community who were not directly involved in healthcare and were unaware of specific osteoporosis cases, thereby minimizing potential biases and ensuring the integrity of the research findings.

The variables included in this study were either independent or dependent. Independent variables included demographics, such as age, sex, educational level, family history of osteoporosis, prior screening or testing for osteoporosis, intention to undergo osteoporosis screening, prior diagnosis with osteoporosis, history of chronic disease, weight (obesity is defined as a body mass index of 30 or higher), current smoking status (who has smoked at least 100 cigarettes in their lifetime and has smoked part or all of the cigarette in the last 28 days), history of prednisolone (steroid) use for more than one month, use of an assistive device to ambulate, calcium and vitamin D intake, physical activity level (any bodily movement produced by skeletal muscle that requires energy expenditure at least 150 minutes throughout the week), history of falls within the past five years, and difficulty in traveling to doctor appointments alone. These factors were examined for their potential associations with the dependent variable, self-efficacy, for performing health-related behaviors.

Data collection from participants utilized a self-administered questionnaire comprising several components: sociodemographic, risk factors for osteoporosis, mobility, falls, knowledge of osteoporosis, prior screening for osteoporosis, intention to do screening, and personal history of osteoporosis. A pre-validated self-efficacy scale was adopted from previous studies, which is composed of 10 items and aims to assess the perceived self-efficacy regarding availing of screening. Each item contains five Likert-scale-type responses, ranging from 1 (strongly disagree) to 5 (strongly agree). The total possible score (the sum of the individual item scores) ranges from 10 to 50 points, with a higher total score indicating a higher level of self-efficacy. The internal consistency in the scale was assessed in the previous study using the Cronbach alpha coefficient, which was found to be 0.87 by Champion et al. [16]. The Arabic version showed good internal consistency with Cronbach’s alpha of 0.88 [17].

The reliability of the questionnaire was verified through a test-retest approach that involved distributing the same survey twice within a two-week period to industry professionals who were not part of the study. This evaluation used a scale-level content validity index based on the average method (S-CVI/Ave), and the resulting average index was calculated to be 0.9, indicating strong content validity [18]. Furthermore, the questionnaire underwent a comprehensive review and received approval from the Faculty of Preventive Medicine, the Institutional Review Board (IRB), General Directorate of Health Affairs in Madinah (IRB log no 23-074).

Data analysis was performed using IBM SPSS Statistics, version 26.0 (IBM Corp., Armonk, NY). Descriptive statistics were used to calculate means and standard deviations (SD) for continuous variables and percentages and frequencies for categorical variables. The 10 items of the self-efficacy of screening to osteoporosis scale were summed up to get the total score. To assess the internal consistency of the self-efficacy of screening for osteoporosis scale, Cronbach’s Alpha was calculated and yielded a satisfactory value of 84.2. To determine the significant predictors of the self-efficacy of screening for osteoporosis, log-linear regression analysis was employed. There was no multicollinearity between the variables in the model. P < 0.05 was considered statistically significant, while P < 0.01 was considered highly significant (HS). Two-tailed tests were assumed throughout the analysis for all statistical tests.

Prior to participating in the study, participants were fully informed about the purpose, procedures, potential risks and benefits, confidentiality measures, and voluntary nature of their involvement. They can ask questions and provide their informed consent to participate. We ensured that participant information remained confidential and protected throughout the study. Any personally identifiable information was anonymized or coded to maintain privacy. Data were securely stored and accessible only to authorized researchers involved in the study. We were obtaining consent from participants to use their data for research purposes, ensuring they understood how their responses were to be used and that their identity was protected. Participants were informed of their right to withdraw from the study at any time without penalty.

## Results

Sociodemographic characteristics of the participants

A total of 342 completed questionnaires were returned. The mean (SD) age was 66.2 ± 4.3 years, ranging from 60 to 79 years. More than half (230, 67.3%) of the respondents were male, had chronic diseases (226, 66.3%), and were smokers 74 (21.8%). Approximately 145 (42.4%) had undergraduate education, 141 (41.2%) had graduate education, and 13 (3.8%) had post-graduate education. More than half (189, 55.3%) reported irregular exercise, 34 (9.9%) exercised regularly, and 119 (34.8%) did not exercise. The mean (SD) weight was 76.2 ± 15 kg, ranging from 43 to 160 kg (Table 1).

**Table 1:** Demographic and background characteristics of the respondents (n = 342) P < 0.001

Characteristics	Frequency	Percent (%)
Gender		
Male	230	67.3
Female	112	32.7
Educational level		
Intermediate education	43	12.6
Undergraduate education	145	42.4
Graduate education	141	41.2
Post-graduate education	13	3.8
Smoking		
Yes	74	21.8
No	266	78.2
Exercises		
Yes, regularly	34	9.9
Yes, but not regularly	189	55.3
No	119	34.8
Chronic disease		
Yes	226	66.3
No	115	33.7

Factors related to osteoporosis risk, ability to screen, and self-efficacy of screening for osteoporosis

The majority of participants did not use prednisolone for a month (252, 74.1%), did not have a family history of osteoporosis (216, 63.2%), had not experienced any falls in the past five years (223, 65.2%), and had not undergone osteoporosis screening (299, 87.4%). The majority reported having sufficient calcium and vitamin D in their diet (231, 67.5%), not using a crutch (206, 60.2%), and having no plans to undergo osteoporosis screening in the next two months (190, 55.6%). Of the participants, 134 (39.2%) reported being unable to go to hospital alone (Table 2). The mean (SD) self-efficacy of screening for osteoporosis score was 37.7 ± 4.7, ranging from 10 to 50. The distribution of the items related to self-efficacy of screening for osteoporosis is shown in Table 3.

**Table 2:** Factors related to osteoporosis risk and ability to screen among the respondents (n = 342)

Characteristics	Frequency	Percent (%)
Have you used prednisolone for a month?		
Yes	88	25.9
No	252	74.1
Do you have enough calcium and vitamin D in your diet?		
Yes	231	67.5
No	111	32.5
Family history of osteoporosis		
Yes	126	36.8
No	216	63.2
Have you fallen for the past 5 years?		
Yes	119	34.8
No	223	65.2
Do you use a crutch?		
Yes	136	39.8
No	206	60.2
Are you able to go to the hospital alone?		
No difficulties	98	28.7
Some difficulties	110	32.2
Not able to go alone	134	39.2
Did you do osteoporosis screening?		
Yes	43	12.6
No	299	87.4
Planning to do osteoporosis screening for the next 2 months?		
Yes	152	44.4
No	190	55.6

**Table 3:** Distribution of the items related to self-efficacy of screening for osteoporosis

Items	Frequency	Percent (%)
1- You can arrange transportation to get osteoporosis screening		
Disagree or strongly disagree	7	2.0
Neutral	44	12.9
Agree and strongly agree	291	85.1
2- You can arrange other things in your life to have an osteoporosis screening		
Disagree or strongly disagree	7	2.0
Neutral	67	19.6
Agree and strongly agree	268	78.4
3- You can talk to people at the osteoporosis center about your concerns		
Disagree or strongly disagree	13	3.8
Neutral	83	24.3
Agree and strongly agree	246	71.9
4- You can get osteoporosis screening even if you are worried		
Disagree or strongly disagree	6	1.8
Neutral	95	27.8
Agree and strongly agree	241	70.5
5- You can get osteoporosis screening even if you don’t know what to expect		
Disagree or strongly disagree	9	2.6
Neutral	94	27.5
Agree and strongly agree	239	69.9
6- You can find a way to pay for osteoporosis screening		
Disagree or strongly disagree	14	4.1
Neutral	191	55.8
Agree and strongly agree	137	40.1
7- You can make an appointment for osteoporosis screening		
Disagree or strongly disagree	6	1.8
Neutral	92	26.9
Agree and strongly agree	244	71.3
8- You know for sure you can get osteoporosis screening if you really want to		
Disagree or strongly disagree	8	2.3
Neutral	136	39.8
Agree and strongly agree	198	57.9
9- You know how to go about getting osteoporosis screening		
Disagree or strongly disagree	4	1.2
Neutral	197	57.6
Agree and strongly agree	141	41.2
10- You can find a place to have osteoporosis screening		
Disagree or strongly disagree	11	3.2
Neutral	147	43.0
Agree and strongly agree	184	53.8

Predictors of self-efficacy for osteoporosis screening in the multivariate analysis

Smokers were more likely to have higher scores in self-efficacy for osteoporosis screening compared to non-smokers (RR = 1.10; 95% CI = 1.01, 1.21). Participants who did osteoporosis screening (RR = 1.12; 95% CI = 1.01, 1.24) and those who were planning to do osteoporosis screening (RR = 1.10; 95% CI = 1.03, 1.19) were more likely to have higher scores in self-efficacy for osteoporosis screening compared to their counterparts (Table 4).

**Table 4:** Predictors of self-efficacy for osteoporosis screening in log-linear regression analysis

Predictors	Categories	Reference group	P-value	Relative risk (RR)	Lower limit (95% CI)	Upper limit (95% CI)
Gender	Male	Female	0.932	0.99	0.92	1.08
Chronic disease	Yes	No	0.390	0.97	0.89	1.05
Have you used prednisolone for a month?	Yes	No	0.952	0.99	0.89	1.11
Smoking	Yes	No	0.036*	1.10	1.01	1.21
Do you have enough calcium and vitamin D in your diet?	Yes	No	0.427	0.97	0.91	1.04
Exercises	Yes, regularly	No	0.169	1.07	0.97	1.18
	Yes, but not regularly	No	0.487	0.97	0.90	1.05
Family history of osteoporosis	Yes	No	0.630	1.02	0.94	1.10
Have you fallen for the past 5 years?	Yes	No	0.460	0.97	0.89	1.05
Do you use a crutch?	Yes	No	0.462	1.06	0.91	1.22
Are you able to go to the hospital alone?	No difficulties	Not able to go alone	0.85	0.98	0.84	1.16
	Some difficulties	Not able to go alone	0.706	1.03	0.89	1.19
Did you do osteoporosis screening?	Yes	No	0.042*	1.12	1.01	1.24
Planning to do osteoporosis screening for the next 2 months	Yes	No	0.009*	1.10	1.03	1.19
Age	Change per year		0.155	1.01	1.00	1.02
Weight	Change per weight unit		0.329	0.99	1.00	1.00
Educational level	Change per educational level		0.114	1.04	0.99	1.08

## Discussion

This study aimed to identify the factors for getting services of osteoporosis screening among people aged 60 years and older in Al-Madina Munawara. The results shed light on the sociodemographic factors of participants, health-related characteristics, and self-efficacy of screening among the participants. The study was conducted on 342 participants with a mean age of about 66 years. They were mainly males, had chronic diseases, had a university educational background, and were non-smokers. Furthermore, more than half of them reported irregular exercise habits.

The factors related to osteoporosis risk and screening self-efficacy among the participants showed several remarkable tendencies. The majority of participants had not gone through osteoporosis screening and did not have a plan to act in the next two months. However, those who had experienced screening or planned to take action demonstrated higher self-efficacy scores for osteoporosis screening. The previous studies illustrated the worth of educational programs and health promotion interventions in enhancing osteoporosis-related knowledge and actions among elderly people. Khazaeian et al. (2021) found that educational interventions are effective in promoting health behaviors and preventive actions related to osteoporosis screening [18]. Moreover, another study conducted in Malaysia by Chan et al. (2019) showed that participants have moderate knowledge and beliefs about osteoporosis, while they have poor adherence to osteoprotective practices [19].

On the other hand, Nayak et al. (2010) identified that older people have some barriers to osteoporosis screening, such as low belief in vulnerability for osteoporosis and a lack of understanding of the relationships between age and osteoporosis susceptibility [20]. Another previous study conducted among Iranian adolescents by Ghelichkhani et al. (2021) revealed that financial status, educational background, and osteoporosis awareness were the main predictors of self-efficacy among participants [21].

Furthermore, other predictors were reported to have an influence on the self-efficacy of participants, and educational interventions have been approved as one of the techniques to improve participants. In one systematic review, it was identified that mailed education, discussion education brochures, telephone counseling, a smartphone app, and in-class discussions are effective methods [22-25]. Moreover, the correlations between the variables studied in a study by Ahn and Oh (2018) indicated that self-efficacy and healthy behaviors, such as performing osteoporosis activity, consuming osteoporosis food, and preventing falls, were correlated [26]. Another study by Tsamlag et al. suggested that osteoporosis-related knowledge is associated with higher scores of self-efficacy among middle-aged and older adults [27], while a study by Shin et al. (2005) highlighted that self-efficacy of osteoporosis is significantly correlated with gender, participants age, marital state, and jobs [28].

Multivariate regression analysis identified various predictors of self-efficacy for osteoporosis screening among elderly people. Particularly, smokers and those who had undergone or have a plan to undertake osteoporosis screening demonstrated higher self-efficacy scores compared to others. These findings indicate that previous exposure to screening or the intention to have screening positively influences people’s confidence in the capability to engage in screening behaviors for osteoporosis. However, the significant differences between participants were related to these three predictors and the CI levels across the value of 1, which could lack the significance of these factors in relation to the level of self-efficacy. On the other hand, a study conducted in China showed that the self-efficacy of exercises is correlated with young age [29], while another study conducted on menopausal women above 45 years old by Janiszewska et al. (2017) showed that a long period of treatment is positively correlated with self-efficacy [30].

Furthermore, the findings of this study emphasize the importance of interpersonal factors in modeling knowledge and behaviors toward osteoporosis screening. Considering the high incidence of osteoporosis in Saudi Arabia, as shown by studies such as Sadat-Ali et al. (2022) and Alharbi et al. (2023), specific measures customized to specific circumstances are essential [6,15]. However, the rate of osteoporosis in Saudi Arabia is high among men compared to women in some regions of the country, and such a study was conducted in the Eastern region [15]; the awareness of this issue is still questionable.

The findings of this study have several implications for healthcare professionals and officials in Al-Madina Munawara health affairs and similar locations. Initially, there was a need for targeted educational interventions aimed at enhancing awareness of osteoporosis risk factors and the significance of screening, especially among elderly people. These interventions ought to make use of socially suitable means of communication and supplies to effectively reach the target population. Moreover, healthcare practitioners should integrate osteoporosis screening and risk assessment into standard clinical practice, specifically for those people with identified risk factors, such as elderly people, chronic illness, and sedentary lifestyles. This could be attained by the implementation of regular screening protocols for osteoporosis and the provision of sufficient training and supplies for healthcare practitioners. Finally, measures to enhance self-efficacy for osteoporosis screening should focus on tackling certain obstacles that were identified in this study, such as transportation problems, problems with money, and a lack of awareness of the process. Techniques to improve self-efficacy might involve giving useful support, enabling utilization of screening services, and delivering personalized guidance and counseling.

This study was limited by a small sample size from the region of Saudi Arabia, which lacks the generalizability of the study findings. Self-variable measurement could influence elderly people’s responses and the accuracy of collected data. Furthermore, the self-efficacy scale was not measured using the suitable scale, which decreases the significance of the findings. It is recommended to establish comprehensive research among a larger sample size across the country to ensure that the influencing factors are appropriately encountered and with interventional study designs to ensure that the improvement measures of self-efficacy are appropriately implemented.

## Conclusions

In conclusion, this study sheds light on the barriers to obtaining osteoporosis screenings among elderly people in Al-Madina Munawara. The participants had a fair level of screening self-efficacy. The smokers and those who had undergone or have a plan to undertake osteoporosis screening demonstrated higher self-efficacy scores compared to others. Progress could be made in decreasing the burden of osteoporosis and enhancing the overall health and well-being of the older population by addressing these issues using specific interventions and policy measures.

Limitations

The limitations of the study included its limited sample size (342) and low response rate relative to the area covered. Thus, the findings should not be generalized. Inherent limitations of cross-sectional studies included non-response from the participants. The reliance on self-reported data introduces the possibility of recall bias or inaccuracies and nonprobability sampling techniques. Future studies are recommended, for instance, in collaboration with government agencies to cover the entire Kingdom of Saudi Arabia. This would provide a more complete scenario regarding osteoporosis screening services among elderly populations. 
